# Targeting SRC to mediate solasonine’s anti-cancer activity in hepatocellular carcinoma and its potential for multi-cancer therapy

**DOI:** 10.3389/fonc.2026.1777213

**Published:** 2026-04-27

**Authors:** Zheng Liu, Xiao Yang Xia, Jia Yao Yang, Ke Nong Li, Jun Lin Hou

**Affiliations:** 1 Department of Integrated Traditional Chinese and Western Medicine, School of Traditional Chinese Medicine, Henan University of Chinese Medicine, Zhengzhou, China; 2 School of Traditional Chinese Medicine, Henan University of Chinese Medicine, Zhengzhou, China

**Keywords:** hepatocellular carcinoma, solasonine, SRC, bibliometric analysis, network pharmacology

## Abstract

**Introduction:**

Cancer remains a leading cause of mortality worldwide, with persistent therapeutic gaps across its heterogeneous subtypes. Solasonine, a steroidal glycoalkaloid from Solanum species, exhibits broad anti-tumor activity, yet its pan-cancer mechanisms of action remain poorly defined.

**Methods:**

A multi-tiered framework was employed: bibliometric analysis mapped the global research landscape; network pharmacology identified core solasonine-cancer target intersections, followed by GO and KEGG pathway enrichment and drug-target-pathway network construction; qRT-PCR, Western blotting, and CCK-8 assays in HCC cell lines experimentally validated the in silico-predicted hub target, SRC, and interrogated downstream signaling.

**Results:**

Bibliometric analysis showed solasonine research concentrated primarily on breast cancer, bladder cancer, and HCC. Network pharmacology identified eight core targets—SRC, EGFR, AKT1, CDH1, TNF, BCL2, ESR1, and STAT3—with SRC as the central network hub. Experimental validation confirmed solasonine suppressed SRC at both transcriptional and translational levels, and SRC overexpression significantly rescued solasonine-induced proliferative inhibition in HCC cells.

**Discussion:**

These integrated findings identify SRC as a critical mechanistic node mediating the pan-cancer anti-tumor effects of solasonine, particularly in HCC, and provide a rational foundation for its further development as a multi-target therapeutic agent.

## Introduction

Globally, cancer poses a major public health challenge, with high incidence and mortality rates (1). A hallmark of cancer is its pronounced heterogeneity, with substantial differences in etiology, pathological features, and therapeutic strategies across tumor types. This heterogeneity poses considerable challenges for the development of broad-spectrum anti-tumor agents (2). Consequently, identifying common molecular targets amenable to modulation across multiple malignancies has developed into a critical direction in oncology research.

Natural products have long been invaluable sources for anti-cancer drug discovery. Among these, solasonine (SS) has garnered significant interest for its broad-spectrum anti-tumor activities (3). Bibliometric analyses indicate that SS exhibits therapeutic effects against several malignancies, including breast cancer (BC), bladder cancer (BLCA), and hepatocellular carcinoma (HCC). However, existing studies have predominantly focused on individual cancer types (4–6), leaving the fundamental question of whether SS exerts its multi-cancer therapeutic effects through a common molecular mechanism largely unresolved. Elucidating such a mechanism is essential not only for understanding the pharmacological basis of SS but also for establishing a theoretical foundation for developing novel broad-spectrum anti-cancer strategies targeting shared pathways.

To address this knowledge gap, the present study first employed bibliometric analysis to consolidate evidence supporting the therapeutic potential of SS against BC, BLCA, and HCC. Subsequently, a network pharmacology approach was utilized to systematically predict common targets of SS across these three malignancies. This analysis identified a core network comprising eight genes, including SRC, EGFR, AKT1,CDH1,TNF, BCL2, ESR1,and STAT3, with SRC hypothesized to occupy a central position within this network. To experimentally validate these predictions, HCC was selected as a model system, and molecular biology and cellular functional assays were conducted to investigate whether SS modulates malignant cell behaviors via SRC. This study aims to delineate the anti-tumor mechanisms of SS from a pan-cancer perspective, thereby providing experimental evidence to support its potential development as a multi-targeted anti-cancer agent.

## Data sources and analytical methods

**Figure 1:**
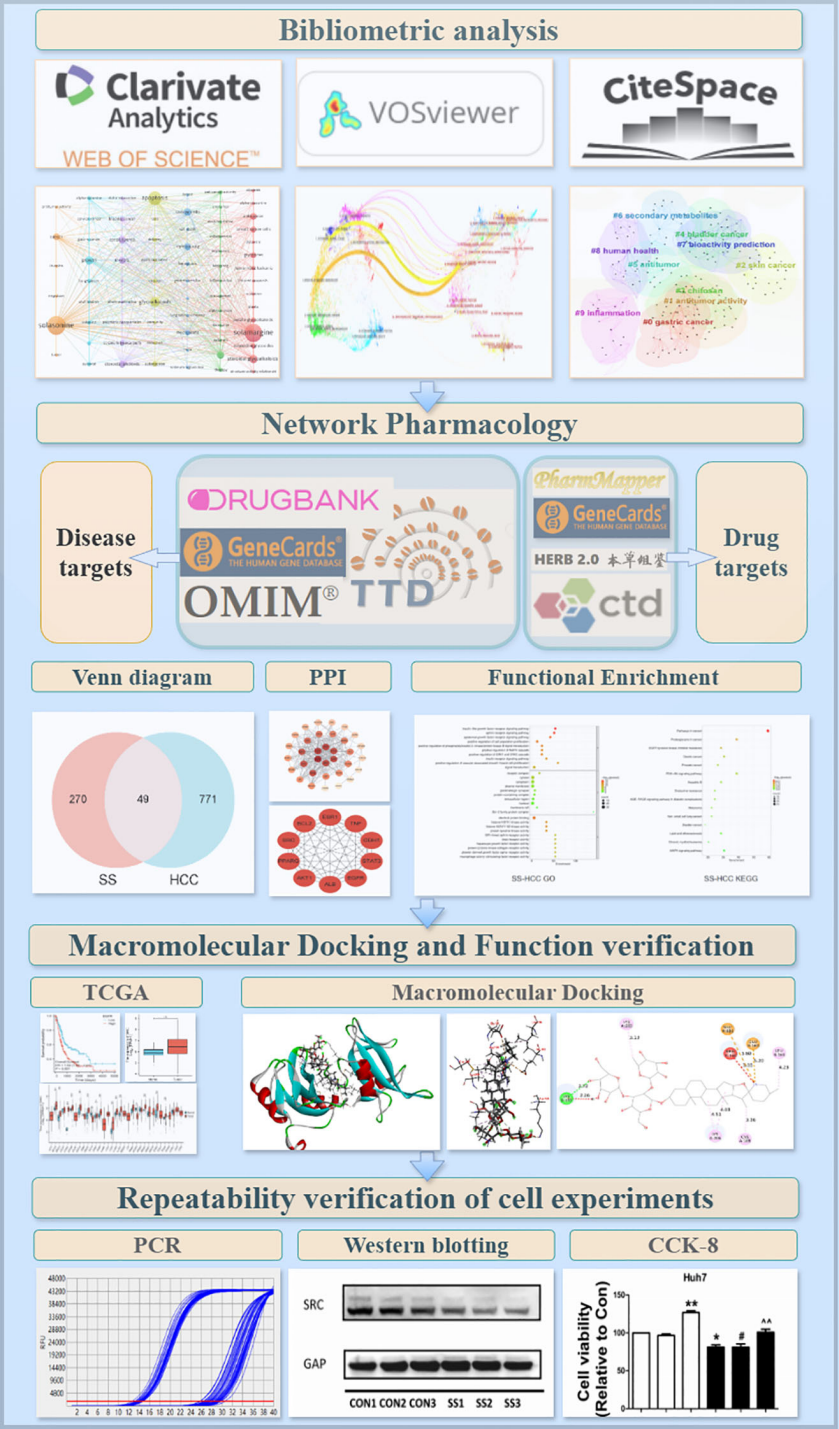
Schematic of the study workflow.

A schematic diagram of the experimental procedure is shown in **Figure 1**.

### Bibliometric analysis of solasonine in cancer treatment

Data from the Web of Science Core Collection were analyzed bibliometrically. The database was queried using the topic search term TS = (solasonine AND cancer), yielding an initial dataset of 48 publications. Following the application of filters for document type (Article or Review) and language (English), along with manual exclusion of irrelevant records, 44 articles were deemed eligible for inclusion. Bibliometric data from these publications were subsequently analyzed and visualized using VOSviewer (version 1.6.10) and CiteSpace (version 6.4.R1) to examine key dimensions, including publication output by country, journal distribution, author contributions, citation networks, and keyword co-occurrence.

### Network pharmacology analysis of potential targets and molecular mechanisms of solasonine in cancer treatment

Solasonine (SS) targets were sourced from the PharmMapper, GeneCards, CTD, and HERB databases. Gene lists retrieved from the four databases were integrated to construct a unified dataset. The identified targets were standardized and validated using the UniProt database. Disease-associated targets for breast cancer (BC), bladder cancer (BLCA), and hepatocellular carcinoma (HCC) were collected from the DrugBank, GeneCards, OMIM, and TTD platforms. Genes retrieved from the four databases were integrated into a combined gene set. Overlapping targets between SS and the three cancer types were identified as candidate targets for subsequent analysis. Common target PPI networks were created using the STRING database and displayed using Cytoscape software (version 3.10.2). Hub targets were identified through topological analysis of node importance using the cytoHubba plugin (degree centrality). The overlapping targets underwent Gene Ontology (GO) enrichment and KEGG pathway analyses through the DAVID bioinformatics platform, with the figures generated using the Microbioinformatics website. Using Cytoscape, a network was built and visualized to integrate multi-dimensional relationships between compounds, targets, and pathways.

### Molecular docking validation of solasonine with key targets

Common targets of SS across BC, BLCA, and HCC were identified via the Venny online tool. The 3D structure of SS was modeled with Chem3D 20.0, while crystal structures of candidate targets were retrieved from the RCSB PDB. Molecular docking analysis was conducted using Discovery Studio 2019 software (BIOVIA, San Diego, CA, USA) to predict the binding mode and affinity between SS and the target protein. The three-dimensional crystal structure of the protein was retrieved from the Protein Data Bank, and the binding site was defined based on the co-crystallized ligand or predicted active cavities. Water molecules and extraneous ligands were removed from the protein structure, followed by the addition of hydrogen atoms. The three-dimensional structure of the ligand SS was energy-minimized and prepared for docking. Docking simulations were performed with default parameters, generating multiple conformational poses. Specific molecular interactions, including hydrogen bonds, hydrophobic contacts, and electrostatic forces, were visualized and analyzed using the visualization tools in Discovery Studio to elucidate the binding mechanism at the atomic level.

### Rescreening of SRC as a priority target

Among the eight overlapping genes, SRC was prioritized for further investigation based on its central topological position within the PPI network and its potential clinical relevance, which was evaluated using the Xiantao Academic online platform. The expression patterns of SRC across pan-cancer datasets were examined using the GEPIA2 database.

### Cell culture and transfection

Huh7 cells were cultured in DMEM supplemented with 10% FBS, 100μg/mL streptomycin, and 100 U/mL penicillin at 37°C under 5% CO2. For transfection experiments, antibiotic-free medium was used to avoid interference with transfection efficiency.

For overexpression studies, Huh7 cells were seeded into 6-well plates at a density of 3×10^5^ cells/well and allowed to reach 50–60% confluence prior to transfection. Cells were then transfected with either the empty vector control plasmid (GV492) or the SRC overexpression plasmid (GV492-SRC-OE), both of which were purchased from GeneChem (Shanghai, China), using Lipofectamine 3000 reagent (Yeasen Biotechnology (Shanghai) Co., Ltd.) at a final plasmid concentration of 2 μg/mL for 24 h at 37°C according to the manufacturer’s instructions.

In parallel, for cell viability assays, Huh7 cells were seeded into 96-well plates at a density of 2× 10^3^ cells/well and transfected with GV492 or GV492-SRC-OE using Lipofectamine 3000 reagent at 0.4 μg/well for 24 h at 37°C, followed by treatment with 30 μM SS for an additional 24 hours.

### Quantitative real-time PCR

Total RNA was extracted using TRIzol reagent (Invitrogen), and RNA concentration and purity were assessed spectrophotometrically. One microgram of total RNA was reverse-transcribed to cDNA using a commercial reverse transcription kit. qRT-PCR was conducted with SYBR Green PCR Master Mix under the following conditions: initial denaturation at 95°C for 30 s, followed by 40 cycles of 95°C for 5 s and 60°C for 30 s. All reactions were performed in triplicate, and relative gene expression was quantified using the 2^(−ΔΔCt) method with GAPDH as the internal control. Primers were synthesized by Sangon Biotech Co., Ltd. (Shanghai, China) with the following sequences:

Gene Direction Sequence (5′–3′)

SRC Forward GAGCGGCTCCAGATTGTCAA

SRC Reverse CTGGGGATGTAGCCTGTCTGT

AKT Forward GTCGCCTGCCCTTCTACAAC

AKT Reverse CACACGATACCGGCAAAGAA

### Western blotting

Huh7 cells were seeded in 6-well plates at a density of 4×10^5^ cells per well and treated with 45μM SS for 24 h. Subsequently, cells were lysed with RIPA buffer supplemented with a protease inhibitor cocktail, and protein concentrations were determined. Equal amounts of protein were mixed with 4×SDS sample buffer, separated on 10% SDS-polyacrylamide gels, and transferred onto polyvinylidene difluoride membranes. The membranes were incubated overnight at 4°C with primary antibodies against SRC (1:1,000) and GAPDH (1:1,000). After washing, the membranes were incubated for 1 h at room temperature with a horseradish peroxidase–conjugated anti-rabbit IgG secondary antibody (Cell Signaling Technology, Beverly, MA, USA). Protein bands were visualized using a freshly prepared enhanced chemiluminescence substrate (Millipore, Burlington, MA, USA) and imaged with a chemiluminescence detection system. Band intensities were quantified using ImageJ software (National Institutes of Health, Bethesda, MD, USA) and normalized to GAPDH expression.

### CCK-8 assay

Huh7 cells were seeded in 96-well plates at 2,000 cells/well and transfected with GV492 or GV492-SRC-OE using Lipofectamine 3000 reagent at 0.4 μg/well for 24 h at 37°C, followed by treatment with 30 μM SS for another 24 h. To evaluate relative cell viability, 10 µL of CCK-8 reagent was added to each well, followed by incubation at 37°C in the dark for 2 hours. Absorbance was then measured at 450 nm using a microplate reader.

## Results

### Bibliometric analysis

A bibliometric analysis was conducted to characterize the evolving research landscape of solasonine in treating cancer. The findings are presented across several dimensions, including country/region contributions, journal citation networks, leading authors, and keyword trends.

### Country/region contributions and collaboration networks

**Figure 2:**
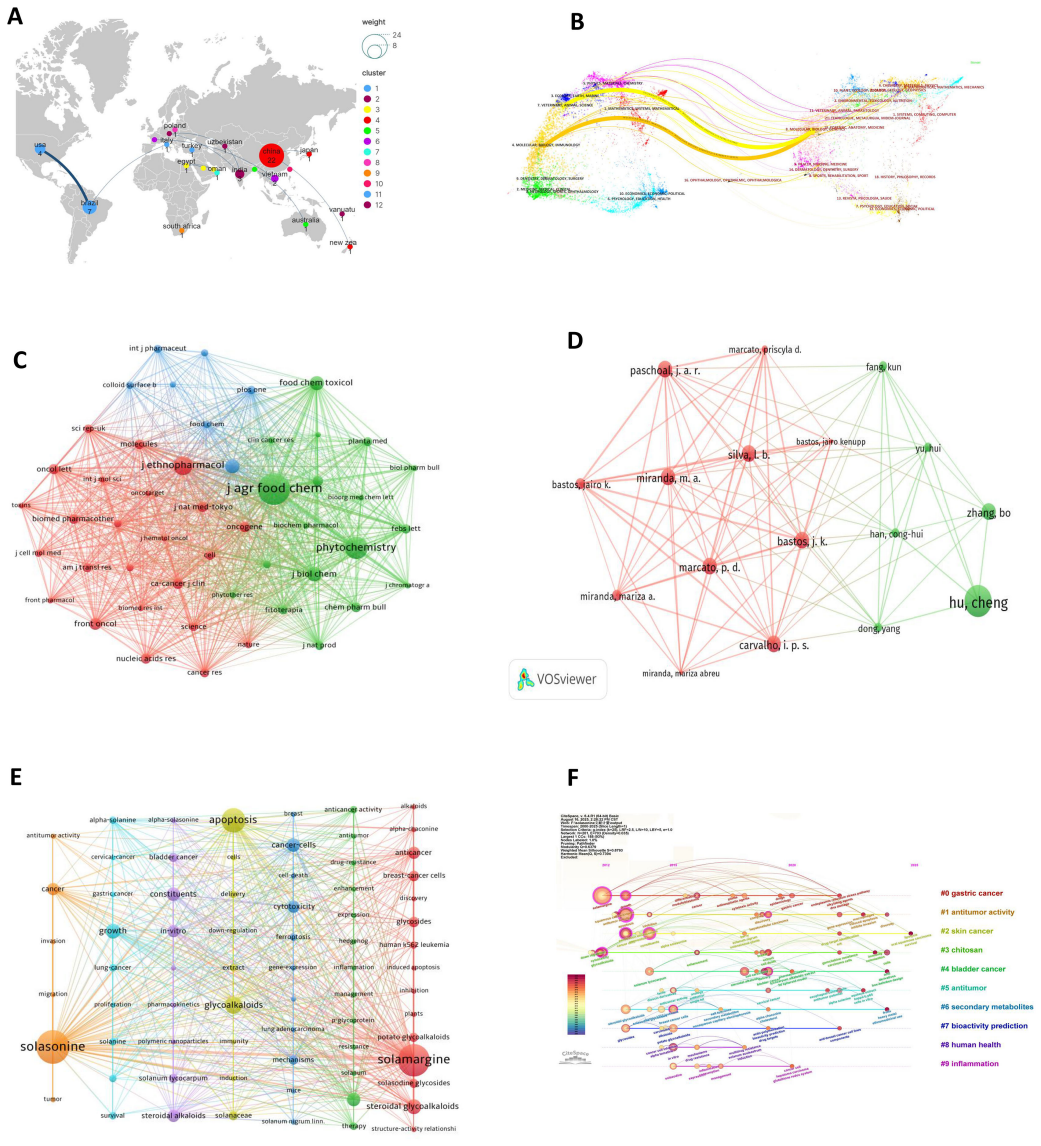
Bibliometric overview of solasonine (SS) in oncology. **(A)** Global collaboration network. **(B)** Journal citation overlay. **(C)** Co-citation network of journals. **(D)** Author co-authorship network. **(E)** Keyword co-occurrence network. **(F)** Temporal evolution of keywords.

The top three countries by publication output in the field of solasonine and cancer research were China (*n* = 22), Brazil (*n* = 7), and the United States (*n* = 4). Collaboration network analysis revealed a strong cooperative relationship between Brazil and the United States (**Figure 2A**).

### Journal co-citation analysis

The journal overlay visualization revealed distinct citation pathways. Two significant paths were identified: a bright yellow path originating from the cited journal cluster “MOLECULAR, BIOLOGY, GENETICS” to the citing journal cluster “VETERINARY, ANIMAL, SCIENCE” (*z* = 2.73791, *f* = 17945), and an orange path from “MOLECULAR, BIOLOGY, GENETICS” to “MOLECULAR, BIOLOGY, IMMUNOLOGY” (*z* = 2.81088, *f* = 18333) (**Figure 2B**). In the journal co-citation network, three journals received more than 40 citations. The most frequently cited was the Journal of Agricultural and Food Chemistry (89 citations; Impact Factor: 6.2; JCR Q1), followed by Phytochemistry (57 citations; IF: 3.4; JCR Q1) and the Journal of Ethnopharmacology (46 citations; IF: 5.4; JCR Q1) (**Figure 2C**).

### Analysis of leading authors

The top 17 authors contributed 37 publications, accounting for 84.1% of the total output. The most prolific author was Hu Cheng (Shanghai University of Traditional Chinese Medicine, China), with four publications and the highest cumulative citation count (149). His research focuses on the mechanisms of bioactive components in traditional Chinese medicine. Kenupp Bastos J (University of São Paulo, Brazil) ranked second in publication number (*n* = 3), with 30 cumulative citations, and primarily investigates the pharmacology and toxicology of natural product extracts. Zhang Bo (Department of Neurosurgery, The Second Affiliated Hospital of Dalian Medical University, China) had the second-highest citation count (55), with research interests centered on the diagnosis and treatment of intracranial tumors (**Figure 2D**). Notably, although China produced the highest number of publications and total citations (496), the United States, with only four publications, accumulated 192 citations, yielding the highest average citation rate per article (48).

### Keyword timeline analysis

A total of 353 keywords were identified. The most frequent keywords were “solasonine,” “solamargine,” and “apoptosis.” Keyword clustering and timeline analyses performed using CiteSpace software identified three cancer types with the highest centrality: “breast cancer cells,” “bladder cancer,” and “hepatocellular carcinoma.” The keyword timeline graph illustrated the first occurrence year of each keyword, indicating that “solasonine” first appeared in 2014 (**Figures 2E, F**; **Table 1**).

**Table 1:** Primary cancer types in SS-focused anti-cancer research.

Cancer	Degree	Centrality
breast cancer cells	24	0.19
bladder cancer	20	0.16
hepatocellular carcinoma	11	0.09
human k562 leukemia	11	0.06
basal cell carcinoma	8	0.02
gastric cancer	6	0
cervical cancer	6	0
cutaneous melanoma	5	0
esophageal cancer cells	5	0
lung cancer	4	0.09

### Network pharmacology analysis

#### Solasonine in breast cancer

**Figure 3:**
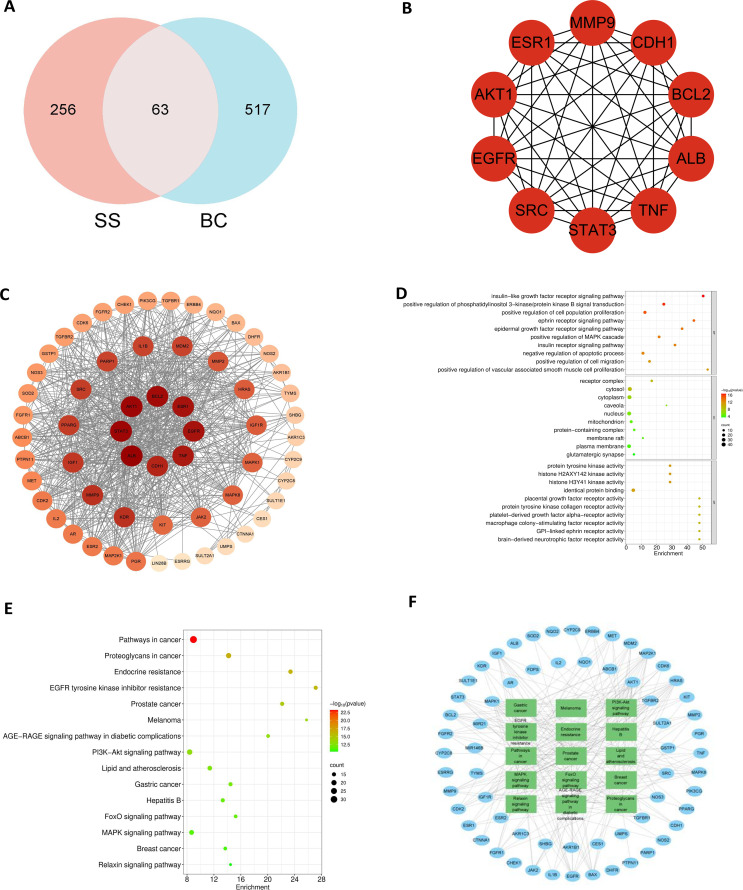
Network pharmacology of SS in breast cancer. **(A)** Venn diagram of intersecting targets. **(B)** Hub target identification. **(C)** PPI network. **(D)** GO enrichment. **(E)** KEGG pathway enrichment. **(F)** Target-pathway network.

Potential targets of solasonine were retrieved from the PharmMapper, GeneCards, CTD, and HERB databases, yielding a total of 319 candidate targets. Separately, known disease-associated targets for breast cancer, bladder cancer, and liver cancer were compiled from four established repositories: DrugBank, GeneCards, OMIM, and the TTD Platform. The intersection of targets across all four databases was taken for each cancer type, resulting in 580 high-confidence targets for breast cancer, 740 common targets for bladder cancer, and 820 targets for liver cancer, respectively. A total of 63 mutual targets for solasonine and breast cancer (BC) were found (**Figure 3A**), which laid the groundwork for the ensuing network pharmacology analysis. With 60 nodes and 724 edges, the PPI network had an average degree of 24.1 per node. Key potential targets—including EGFR, MMP9, SRC, ALB, BCL2, CDH1, AKT1, TNF, ESR1, and STAT3—were identified as potentially central to solasonine’s anti-BC mechanisms (**Figures 3B, C**).

GO enrichment analysis of the top 10 terms demonstrated that solasonine may influence biological processes (BP) such as protein tyrosine kinase activity, histone H2AXY142 and H3Y41 kinase activities, identical protein binding, and placental growth factor receptor activity. Affected cellular components (CC) included the receptor complex, cytosol, cytoplasm, caveola, and nucleus. Relevant molecular functions (MF) involved the IGFR signaling pathway, positive regulation of the PI3K-AKT pathway, cell population proliferation, ephrin and epidermal growth factor receptor signaling pathways (**Figure 3D**). KEGG pathway analysis further suggested that solasonine may modulate BC progression primarily via pathways like the PI3K-AKT, FoxO, and MAPK signaling pathways (**Figure 3E**). The target–pathway network illustrated that solasonine exhibits anti-BC efficacy through a multifaceted mechanism involving multiple targets and pathways, with enrichment in pathways related to gastric cancer, tyrosine kinase inhibitor resistance, among others (**Figure 3F**).

#### Solasonine in bladder cancer

**Figure 4:**
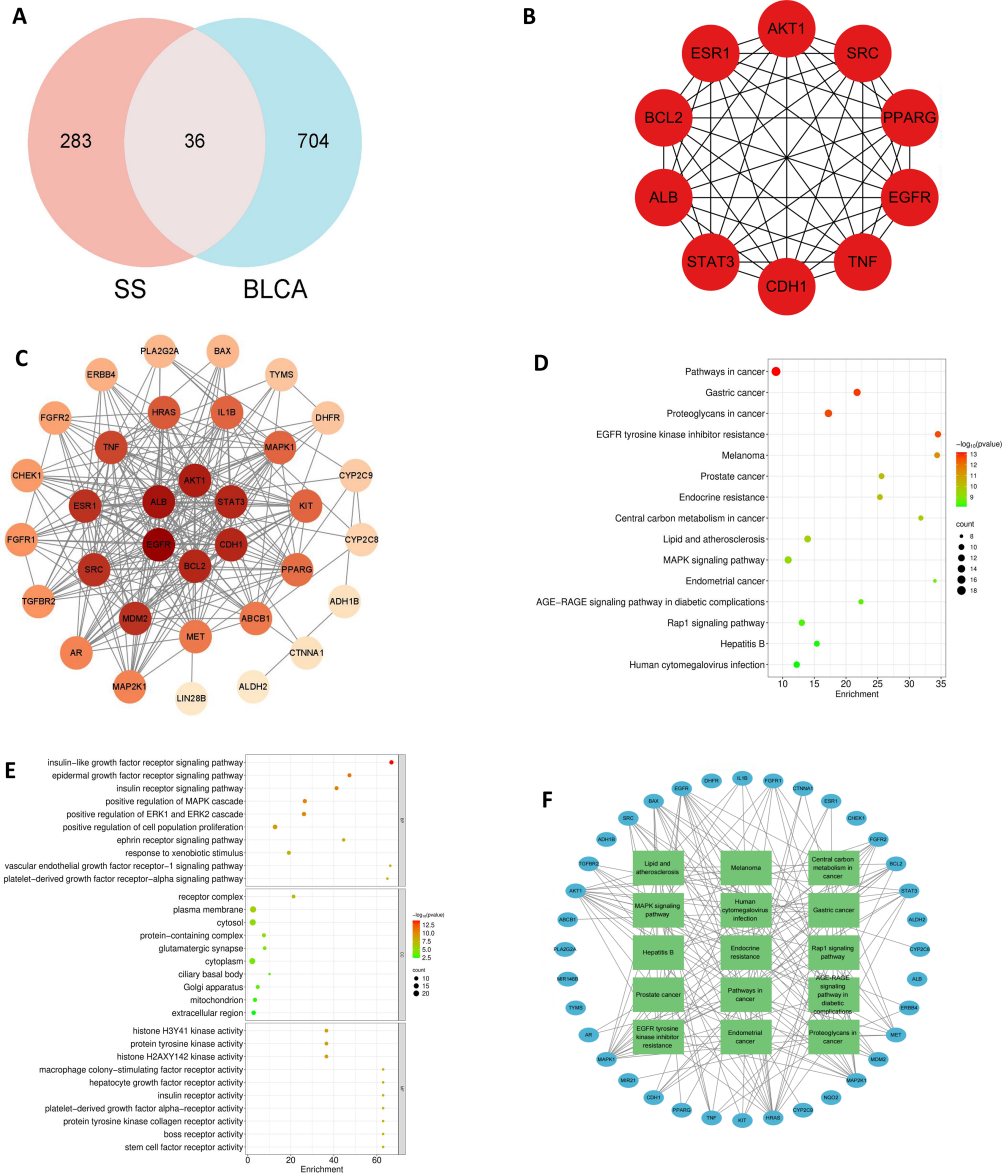
Network pharmacology of SS in bladder cancer. **(A)** Venn diagram of overlapping targets. **(B)** Core therapeutic targets. **(C)** PPI network. **(D)** KEGG enrichment analysis. **(E)** GO annotation **(F)** Target-pathway mapping.

Analysis via network pharmacology yielded 36 shared targets between solasonine and BLCA (**Figure 4A**). The resultant PPI network (34 nodes, 247 edges) exhibited a high connectivity density, reflected by an average node degree of 14.5 (**Figure 4C**). Among these, EGFR, STAT3, CDH1, TNF, ESR1, BCL2, SRC, PPARG, AKT1, and ALB emerged as potentially critical mediators of solasonine’s actions in BLCA (**Figure 4B**).

GO term analysis indicated enrichment in biological processes including histone H3Y41 and H2AXY142 kinase activities, protein tyrosine kinase activity, and macrophage colony-stimulating factor and hepatocyte growth factor receptor activities. Key cellular components impacted were the receptor complex, plasma membrane, cytosol, protein-containing complex, and glutamatergic synapse. Significantly enriched molecular functions encompassed insulin-like growth factor, epidermal growth factor, and insulin receptor signaling pathways, as well as positive regulation of the MAPK and ERK1/2 cascades (**Figure 4E**). KEGG pathway enrichment analysis highlighted pathways such as central carbon metabolism in cancer, lipid and atherosclerosis, MAPK, and Rap1 signaling. (**Figure 4D**). The target–pathway network supports a multi-target mechanism of solasonine in bladder cancer suppression, with enrichment in pathways including MAPK signaling, melanoma, and hepatitis B, et al. (**Figure 4F**).

#### Solasonine in hepatocellular carcinoma

**Figure 5:**
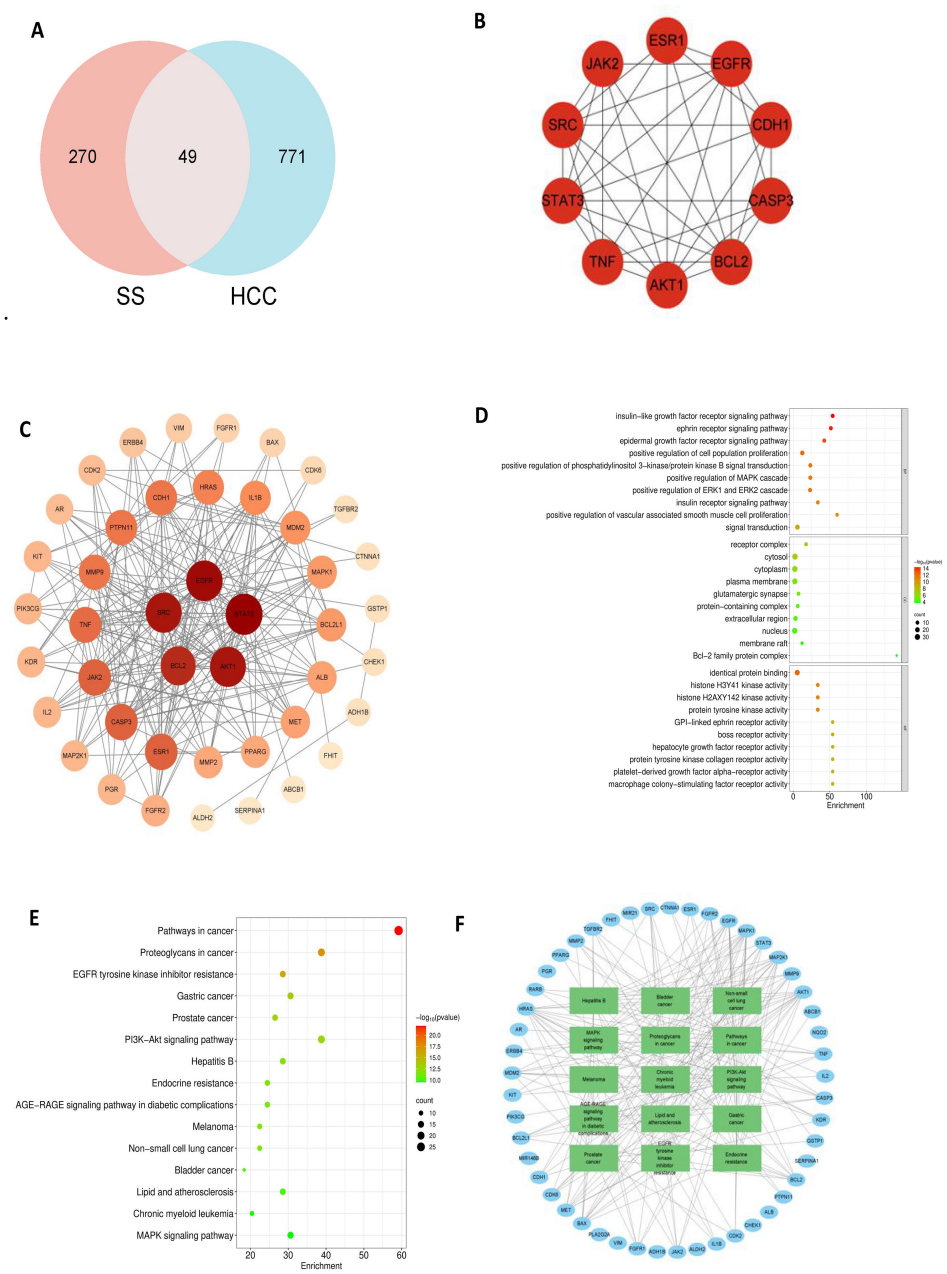
Network pharmacology of SS in hepatocellular carcinoma. **(A)** Venn diagram of common targets. **(B)** Key target proteins. **(C)** PPI network. **(D)** GO analysis. **(E)** KEGG pathway analysis. **(F)** Integrated target-pathway network.

For hepatocellular carcinoma (HCC), 49 shared targets between solasonine and HCC were retrieved for network pharmacology evaluation (**Figure 5A**). The resulting PPI network consisted of 44 nodes and 230 edges, exhibiting an average node degree of 10.5(**Figure 5C**). Core targets, including AKT1, BCL2, CASP3, CDH1, EGFR, ESR1, JAK2, SRC, STAT3, and TNF, were implicated in solasonine’s potential anti-HCC effects (**Figure 5B**). GO analysis revealed solasonine’s involvement in biological processes including identical protein binding, histone kinase activities (H3Y41 and H2AXY142), protein tyrosine kinase activity, and GPI-linked ephrin receptor activity. Cellular components affected included the receptor complex, cytosol, cytoplasm, plasma membrane, and glutamatergic synapse. Molecular functions featured insulin-like growth factor receptor, ephrin receptor, and epidermal growth factor receptor signaling pathways, along with positive regulation of cell proliferation and PI3K-AKT signaling (**Figure 5D**). KEGG pathway analysis indicated putative roles in the PI3K-AKT, MAPK, and lipid and atherosclerosis pathways (**Figure 5E**). The constructed target–pathway network illustrates solasonine’s multi-target inhibitory action against HCC, with enrichment in pathways including MAPK signaling, melanoma, and hepatitis B, et al. (**Figure 5F**).

The fourth and fifth most prevalent tumor types in solasonine-focused anti-cancer research were human K562 leukemia (CML) and basal cell carcinoma (BCC), the relevant findings are presented in the Supplementary Materials.

### Molecular docking validation of solasonine with key targets

**Figure 6:**
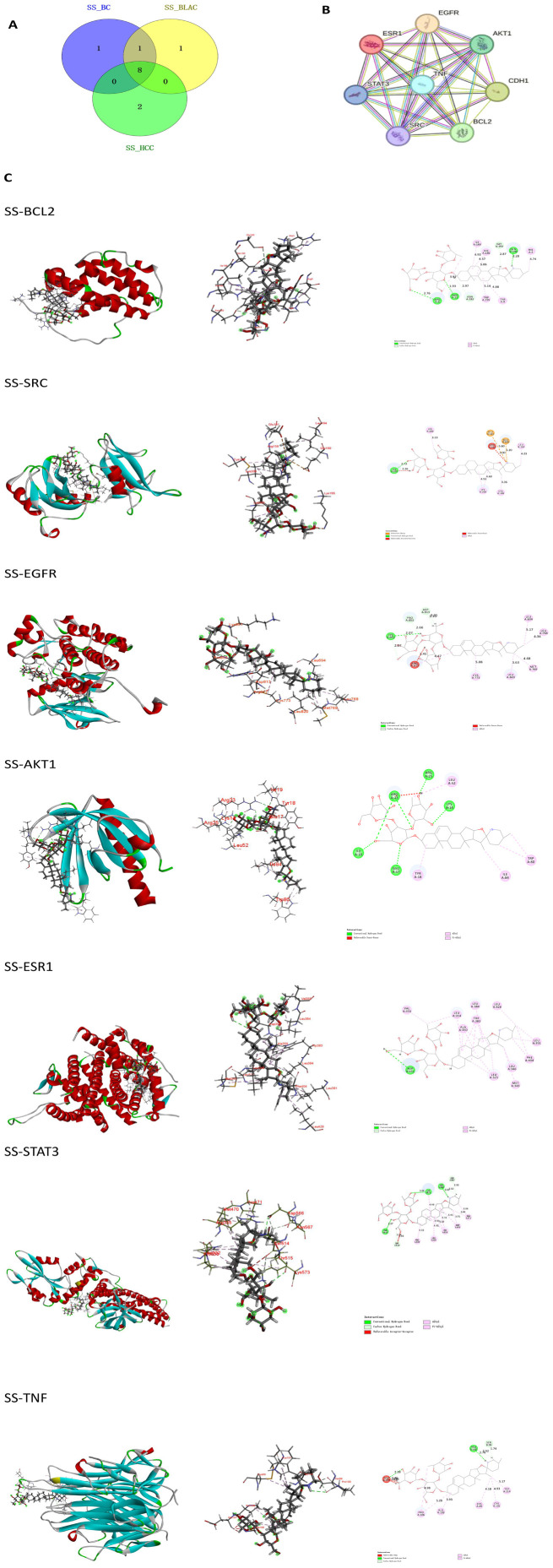
Inhibition of shared core targets by SS across three cancers. **(A)** Overlap of the top 10 core targets in BC, BLCA, and HCC. **(B)** PPI network of the 8 shared hub proteins. **(C)** Molecular docking poses of SS with core targets.

The analysis of network pharmacology identified targets and pathways that solasonine might use to regulate BC, BLCA, and HCC cells. By intersecting the core target genes associated with solasonine’s inhibitory effects on BC, BLCA, and HCC, we obtained a gene set concurrently implicated in all three cancer types–SRC, BCL2, CDH1, AKT1, EGFR, ESR1, STAT3, TNF (**Figures 6A, B**). To analyze the binding sites and interaction modes, Discovery Studio was employed, and the results revealed that SS binds to the active site of SRC primarily through alkyl interactions with residues LYS203, LYS206, CYS188, and LEU164; attractive charge interactions with GLU181 and GLU160; unfavorable donor–donor or positive–positive contacts with ARG158 and LYS155; and a conventional hydrogen bond with LYS155. Together, these interactions contribute to stable binding. The binding sites and interaction patterns for the other molecules are summarized in **Table 2** and **Figure 6C**.

**Table 2:** Molecular docking analysis of the binding sites and modes for SS with target proteins.

Protein	Binding sites	Binding modes
BCL2	ARG6, ASN11,ASN182,TRP195,TYR9,ILE189, HIS186, HIS3, GLN190, GLY193	Alkyl and Pi-Alkyl: ARG6, ILE189, HIS186, HIS3, TRP195 and TYR9;conventional Hydrogen Bond: ARG6, ASN11, GLN190; carbon Hydrogen Bond: ASN182, GLY193
SRC	LYS203, LYS206,CYS188,LEU164,GLU181,GLU160,ARG158 and LYS155	Alkyl: LYS203, LYS206,CYS188,LEU164, attractive Charge: GLU181, GLU160, unfavorable donor-donor unfavorable positive-positive: ARG158 and LYS155. Conventional hydrogen bond: LYS155
EGFR	LYS851,ARG817,PRO853,ASP813,LEU694,LEU768,MET769,LEU820,CYS773	Alkyl: LEU694,LEU768,MET769,LEU820,CYS773,ARG817; carbon hydrogen bond: LYS851,PRO853,ASP813; Conventional hydrogen bond: LYS851;Unfavorable Donor-Donor: ARG817
AKT	ILE19,GLU17,TYR18,ILE84,TRP80,LYS14,LEU52,ARG25,ARG23	Alkyl or Pi-Alkyl: LEU52,TRP80,TYR18,ILE84; Conventional hydrogen bond: ILE19, GLU17, ARG25, ARG23,LYS14;Unfavorable Donor-Donor: ARG23
ESR1	VAL355,LEU354,ALA350,TRP383,LEU384,LEU428,LEU391,PHE404,LEU346,MET343,LEU525,ASP351	Alkyl or Pi-Alkyl: VAL355,LEU354,ALA350,TRP383,LEU384,LEU428,LEU391,PHE404,LEU346,MET343,LEU525; Conventional hydrogen bond and carbon hydrogen bond: ASP351
TNF	GLU107,PRO100,SER99,TRP114,CYS101,CYS69,ALA109,PRO106	Alkyl or Pi-Alkyl: TRP114,CYS101,CYS69,ALA109,PRO106; Conventional hydrogen bond PRO100,GLU107;unfavorable bump: GLU107;carbon hydrogen bond SER99,PRO100
STAT3	THR515,SER514,PRO333,ARG335,HIS332,MET470,PEO471,LYS573,ASP566,ASN567	Alkyl or Pi-Alkyl: PRO333,ARG335,HIS332,MET470,PEO471,LYS573,Conventiona hydrogen bond LYS573,ASP566,THR515;carbon hydrogen bond ASP566,ASN567,unfavorable acceptor-acceptor SER514

### Screening and validation of SRC expression in hepatocellular carcinoma

**Figure 7:**
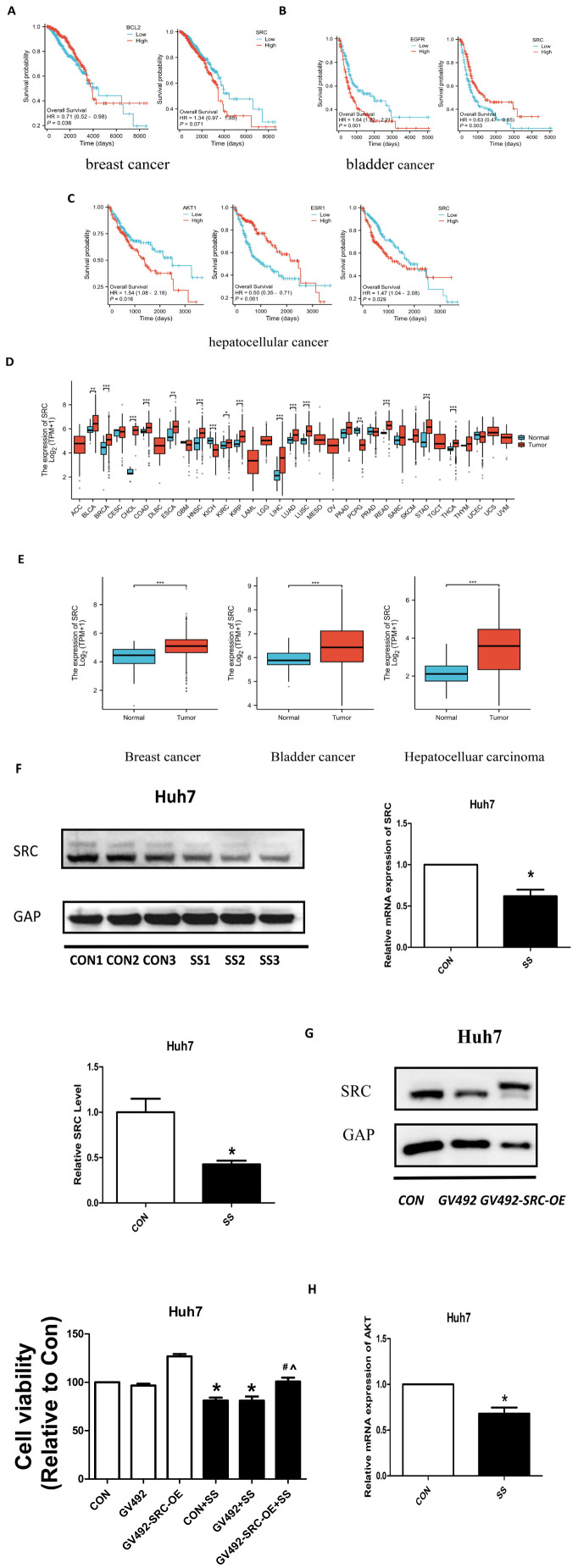
SS inhibits hepatocellular carcinoma by targeting SRC. **(A–C)** Prognostic value (overall survival) of core targets. **(D)** Pan-cancer expression patterns of SRC. **(E)** SRC expression in BC, BLCA, HCC. **(F)** SS downregulates SRC expression in HCC cells. **(G)** SRC overexpression rescues the anti-proliferative effect of SS. **(H)** SS downregulates AKT mRNA expression. * indicates *P*<0.05; ** indicates *P*<0.01; *** indicates *P*<0.001; # indicates *P*<0.05 compared with the con+SS group; ^ indicates *P*<0.05 compared with the GV492+SS group.

Using the Xiantao Academic online platform, we evaluated the prognostic impact of the eight core genes on overall survival (OS) in BC, BLCA, and HCC. The research results have been released. Apart from BLC2, SRC, EGFR, AKT1, ESR1, the rest of the genes are not related to prognosis. In breast cancer, BCL2 expression was markedly correlated with prognosis (*P* < 0.05), whereas SRC exhibited a trend toward significance (*P* = 0.071; **Figure 7A**). In contrast, SRC expression was significantly correlated with prognosis in both bladder cancer and hepatocellular carcinoma (*P* < 0.05; **Figures 7B, C**). Based on these findings, SRC was prioritized for further investigation. Analysis of public datasets revealed that SRC is expressed at elevated levels across multiple tumor types, including BC, BLCA, and HCC (**Figures 7D, E**). Subsequent experimental validation by qRT-PCR and Western blotting confirmed that solasonine treatment significantly downregulated SRC expression in hepatocellular carcinoma cells at both the transcriptional and translational levels (**Figure 7F**).

### SRC overexpression rescues solasonine-induced suppression of proliferation in HCC cells

To further establish SRC as a crucial functional target of solasonine, rescue experiments were performed in hepatocellular carcinoma cells. It was foreseen that solasonine would effectively restrict the proliferation of HCC cells. However, upon ectopic overexpression of SRC, the anti-tumor effects of solasonine were markedly attenuated. The effect of solasonine in inhibiting cell proliferation was considerably diminished (**Figure 7G**). In addition, The expression of AKT mRNA, a downstream target of SRC, was observed downregulation. This suggests that SS may inhibit hepatocellular carcinoma growth by regulating downstream AKT1 expression through SRC (**Figure 7H**).

## Discussion

*Solanum nigrum L*. has a centuries-long history of medicinal use, and its anticancer properties have been extensively documented. Solasonine (SS), a major steroidal glycoalkaloid isolated from Solanum species, exhibits broad-spectrum antitumor activity, with particular efficacy demonstrated against HCC cells (3). Nevertheless, its precise therapeutic mechanisms across different cancer types remain incompletely elucidated and warrant systematic investigation.

By employing an integrative strategy that includes bibliometric analysis, network pharmacology, molecular docking, and experimental validation, the present study provides compelling evidence that solasonine (SS) exerts anti-tumor effects across multiple cancers—including BC, BLCA, and HCC—by targeting a shared core network of signaling molecules, with the non-receptor tyrosine kinase SRC serving as a pivotal mediator.

Bibliometrics is a citation analysis-based methodology. It enables systematic mapping of research landscapes, identification of hotspots, and forecasting of developmental trajectories. Applying this approach to the global SS antitumor literature, we identified several structurally coherent and mechanistically meaningful patterns.

China dominates SS antitumor research, reflecting both the deep-rooted ethnopharmacological legacy of *Solanum nigrum L.* in traditional Chinese medicine and the country’s disproportionately high burden of HCC and BC (7–9). Besides, SS is predominantly co-investigated with solamargine (SM), both being steroidal glycoalkaloids derived from *Solanum nigrum L.* that share the same aglycone, yet confer complementary cytotoxic profiles (10); Keyword analysis identifies apoptosis as the dominant mechanistic theme, whereby SS activates the intrinsic cascade via Bax/Bcl-2 modulation and caspase-3 activation (4), and disrupts mortalin–p53 interaction to induce apoptosis in both TP53-proficient and -deficient HCC cells (5). Notably, SS also induces ferroptosis by suppressing GPX4 and GSS, validated in HCC cancer models (11), representing an emerging mechanistic frontier.

Moreover, our analysis identifies bladder cancer (BLCA), breast cancer (BC), and hepatocellular carcinoma (HCC) as the three predominant tumor types in the SS literature. In BLCA, SS directly binds the b1 domain of neuropilin-1 (NRP1), suppressing its protein stability and disrupting the NRP1/VEGFA/VEGFR2 ternary complex and NRP1/EGFR complexes, thereby inhibiting the PI3K/AKT, ERK/MAPK, and P38/MAPK signaling axes, inducing apoptosis and inhibiting proliferation in BLCA cells (12). In BC, SS could inactivate the ERK2/MAPK pathway, thereby inducing ferroptosis and further inhibiting BC cell proliferation, migration, and invasion (6). In HCC, SS exerts its anti-hepatocellular carcinoma (HCC) effects by upregulating miR-375-3p and suppressing lncRNA CCAT1, thereby facilitating SP1-mediated transcriptional repression of IRF5. The delineated CCAT1/miR-375-3p/SP1/IRF5 regulatory axis establishes a novel molecular mechanism underlying SS-induced HCC growth inhibition (13). This tripartite focus reflects an alignment between SS’s broad mechanistic repertoire and the substantial unmet clinical need across these three malignancies.

Network pharmacological analysis identified eight hub target genes mediating the simultaneous antitumor activity of SS across BLCA, BC, and HCC: SRC, STAT3, EGFR, AKT1, BCL2, CDH1, ESR1, and TNF. These targets do not operate in isolation but rather function in a coordinated and interdependent manner, engaging in cooperative cross-talk to collectively orchestrate the underlying biological processes. They represent an interconnected signaling hub through which SS exerts pleiotropic anticancer effects. For example, Da-Chai-Hu-Tang Formula suppresses HepG2 cell proliferation and metastatic potential via PI3K/AKT/STAT3 pathway-mediated induction of cell cycle arrest and apoptosis (14). And, re-expression of ERRα, driven by the cholesterol/EGFR/Src/Erk/SP1 signaling cascade, confers survival advantage in gefitinib- and osimertinib-resistant non-small cell lung cancer cells (15). KEGG and GO enrichment analyses revealed consistent pathway activation across all three tumor types, including endocrine resistance, EGFR tyrosine kinase inhibitor resistance, the AGE-RAGE signaling pathway in diabetic complications, lipid metabolism and atherosclerosis, and the MAPK signaling pathway. The convergent enrichment of these pathways — spanning resistance mechanisms, metabolic reprogramming, and core proliferative cascades — underscores that SS acts upon fundamental hallmarks of cancer biology rather than tumor-type-specific vulnerabilities, providing a compelling mechanistic basis for multi-cancer therapeutic application.

Among the eight identified hub genes, SRC exhibited statistically robust prognostic associations across both BLCA and HCC in transcriptomic survival analyses. High SRC expression was associated with poor prognosis in HCC, whereas low SRC expression correlated with worse prognosis in BLCA. These divergent prognostic patterns suggest that SRC may fulfill distinct, context-dependent roles across tumor types. In HCC, SRC likely functions as a canonical oncogene, whereby its upregulation drives tumor progression and consequently confers unfavorable clinical outcomes. Conversely, in BLCA, despite similarly elevated expression levels, SRC may paradoxically exert context-dependent or pathway-specific effects.

In HCC, SRC predominantly exerts oncogenic functions through multiple converging mechanisms. Specifically, CYP2A6 physically interacts with SRC to suppress the SRC/Wnt/β-catenin signaling axis, thereby attenuating its downstream tumorigenic transcriptional program and producing anti-tumor effects (16). Concurrently, FGF19-activated FGFR4 assembles an endosomal complex with SRC and STAT3 that translocates to the nucleus to sustain oncogenic transcriptional output (17). However, SRC may show context-dependent or pathway-specific effects in BLCA. For example, both Cav-1 depletion and forced expression of active SRC in metastatic UMUC-3 cells attenuate actin stress fiber formation, suppress cell migration, and reduce metastatic burden *in vivo*, whereas Cav-1 overexpression or SRC depletion markedly enhances the migratory potential of non-metastatic RT4 cells (18). Another study demonstrated that RhoGDI2 functions as a bona fide metastasis suppressor in human bladder cancer, and that SRC-mediated phosphorylation of RhoGDI2 potentiates its metastasis-suppressive activity, whereas loss of SRC expression abrogates this suppressive function in tumor cells retaining RhoGDI2 expression (19).

Although SRC may play distinct, context-dependent roles across different tumor types, this divergent prognostic impact neither undermines its validity as a therapeutic target for SS, nor compromises the antitumor mechanism mediated through SS modulation of SRC activity. In HCC, SS is proposed to exert tumor-suppressive effects by transcriptionally downregulating SRC expression, thereby attenuating its oncogenic output. In BLCA, SS may conversely exert its effects by upregulating SRC expression, potentially reinforcing its tumor-suppressive function within this cellular context. Taken together, the prognostic robustness of SRC, combined with its topologically central position within the inferred protein–protein interaction network, nominated it as the most therapeutically actionable candidate warranting in-depth experimental validation.

SRC (proto-oncogene tyrosine-protein kinase Src, c-Src), the first proto-oncogene to be described and characterized, is the founding member of the Src family kinases (SFKs). The SRC protein is organized into SH3, SH2, and kinase (SH1) domains flanked by unique N-terminal and C-terminal regulatory regions. Enzymatic activity is regulated by two critical phosphorylation events: an activating phosphorylation at Tyr419 within the kinase domain, mediated by an adjacent Src molecule in trans, and an inhibitory phosphorylation at Tyr530 located in the regulatory tail, catalyzed by C-terminal Src kinase (Csk) or its homolog Csk homologous kinase (Chk) (20). Upon oncogenic activation, c-Src aberrantly transactivates a spectrum of downstream signaling cascades, encompassing the PI3K/AKT pathway, the Ras/MAPK pathway, the JAK/STAT3 pathway, and the FAK/Paxillin axis. Dysregulation of these interconnected signaling networks has been critically implicated in the promotion of tumor cell proliferation, evasion of apoptosis, enhanced migratory and invasive capacity, metastatic dissemination, and the acquisition of therapeutic resistance (21). Establishing it as a pan-cancer therapeutic target.

Analysis of the SS-SRC docking model identified critical interactions comprising alkyl bonds (LYS203, LYS206, CYS188, LEU164), attractive charge (GLU181, GLU160), a conventional hydrogen bond, and unfavorable contacts (ARG158, LYS155) with functionally important residues. The molecular docking results indicate that SS forms a stable complex with the ATP-binding pocket of SRC kinase through multi-modal interactions, suggesting its potential role as a type II kinase inhibitor. The key binding residues are located within functionally critical regions of SRC: the salt bridge between LYS203 (equivalent to the conserved Lys295) and GLU181 (Glu283) is essential for maintaining the active kinase conformation (22); the interaction with CYS188 (Cys277) may involve allosteric regulation (23); and the non-canonical interactions with ARG158 and LYS155 near the DFG motif are characteristic of stabilizing the inactive “DFG-out” conformation (24). This binding pattern provides a structural rationale for the potent inhibition of SRC by SS.

The oncogenic role of SRC in HCC is well-established and multi-dimensional. Early seminal work demonstrated that pp60c-src kinase activity is markedly elevated in HCC tumor tissue compared with adjacent non-tumorous liver parenchyma, and is essentially undetectable in normal or chronic hepatitis liver tissue; moreover, kinase activity correlates positively with histopathological dedifferentiation grade, suggesting a role in malignant transformation (25). Subsequent immunohistochemical analysis of 52 HCC patients confirmed that both total SRC (t-Src) and its activated phosphorylated form (p-Y416Src) are significantly overexpressed in HCC tissue relative to adjacent non-tumor tissue, with t-Src expression positively correlating with tumor stage, poor cellular differentiation, and metastatic status (26). Mechanistically, CYP2A6 physically interacts with SRC, thereby suppressing the oncogenic SRC/Wnt/β-catenin signaling axis and attenuating its downstream tumorigenic transcriptional program, ultimately exerting potent anti-tumor effects in hepatocellular carcinoma (16); In parallel, FGFR4 activated by FGF19 formed an endosomal complex with Src and STAT3 and moved to the nucleus. Beyond its oncogenic signaling roles, SRC upregulation has been identified as a critical mechanism of acquired resistance to lenvatinib in HCC, where inhibition of SRC with dasatinib partially restored drug sensitivity (17, 27). Furthermore, in a comprehensive preclinical study, SRC and YES1 were found to be significantly co-upregulated in clinical HCC specimens relative to adjacent non-tumoral tissue (*p* < 0.001), with high SRC expression independently associated with poor prognosis in the TCGA-HCC cohort. Dual inhibition of SFKs with sorafenib produced synergistic antitumor effects in multiple HCC cellular models (28). These findings collectively establish SRC as a key driver of HCC progression and a rational therapeutic target.

In BC, SRC functions as a central coordinator of oncogenic signaling across distinct molecular subtypes. At the mechanistic level, SRC directly phosphorylates and co-activates STAT3 and STAT5, with subsequent nuclear translocation of STAT3/5 driving tumor cell proliferation and survival (29, 30). In the context of chemotherapy exposure, SRC-dependent STAT3 activation has been shown to sustain the expression of pluripotency-associated factors and promote breast cancer stem cell (BCSC) enrichment, thereby providing a mechanistic rationale for chemotherapy-induced acquired resistance (31). In TNBC — a subtype characterized by the absence of approved targeted therapies and the highest unmet clinical need — SRC family kinases are transcriptionally upregulated in drug-tolerant persister (DTP) cells. This upregulation establishes a hyperactivated EGFR–SRC signaling node that drives constitutive phosphorylation of STAT3, AKT, and MAPK, ultimately sustaining cell survival under chemotherapeutic pressure (32). In the HER2-positive subtype, elevated pSrc-Y416 levels have been detected in trastuzumab-refractory cells and show significant positive associations with tumor size, necrosis, central nervous system metastasis, p53 overexpression, and MAPK pathway activation, implicating SRC as a key effector of trastuzumab resistance (33). Collectively, these findings position SRC as a critical oncogenic kinase with broad relevance across BC subtypes, particularly in the setting of therapeutic resistance.

In BLCA, multi-omics analyses have demonstrated that SRC expression carries significant prognostic value. Using TCGA-BLCA cohort data, decreased SRC expression was shown to significantly correlate with poorer overall survival in male (*p* < 0.001, HR = 0.45) (34). Mechanistically, SRC cooperates with focal adhesion kinase (FAK) to regulate TGF-β-induced migration and invasion of bladder cancer cells via E-cadherin modulation, thereby facilitating epithelial-to-mesenchymal transition (EMT) and metastatic dissemination (35). Given that BLCA is characterized by a high recurrence rate exceeding 80% in non-muscle-invasive disease and rapid progression to metastasis in muscle-invasive disease (36), the identification of SRC as a prognostically relevant kinase in this context further supports its nomination as a high-value therapeutic target.

Our experimental results demonstrate that SS downregulates SRC mRNA and protein expression in HCC cells, resulting in significant inhibition of hepatocellular proliferation. This finding constitutes the first direct experimental evidence linking the anticancer activity of SS to SRC suppression in HCC, and substantiates the network pharmacological prediction that SRC is a central mediator of SS’s therapeutic effect. The mechanistic coherence is compelling: SRC is a convergent upstream regulator of multiple SS-perturbed pathways — including PI3K/AKT, MAPK, and STAT3 — that were enriched in our KEGG and GO analyses across all three tumor types. Notably, our experiments also demonstrated that SS treatment markedly reduces AKT expression. AKT, a core component of the PI3K/AKT/mTOR signaling axis, is frequently hyperactivated in HCC and closely associated with tumor progression, proliferation, and survival (37). Accumulating evidence indicates that inhibiting AKT activity effectively suppresses HCC development (38). Given that AKT is a well-established downstream effector of SRC, and SRC can phosphorylate and activate upstream components such as PI3K to drive AKT signaling (39), our data support a plausible mechanistic cascade: SS likely inhibits HCC growth by transcriptionally suppressing SRC expression, thereby attenuating its activation of the PI3K/AKT pathway and ultimately reducing both AKT expression and activity. Future studies employing rescue experiments (e.g., overexpression of SRC or constitutively active AKT in the presence of SS) could further establish the causal necessity of this axis and elucidate its detailed regulation of downstream cell-cycle and apoptosis related molecules.

This study elucidates the mechanism by which SS exerts anti-tumor effects across multiple cancer types by regulating SRC expression, thereby deepening our understanding of the intricate molecular actions of SS. These findings position SS as a potential therapeutic strategy for a broad range of cancer patients, including those who have developed resistance to conventional chemotherapy or targeted therapies. Notably, SS targets fundamental hallmarks of cancer biology—exemplified by its action on SRC—rather than tumor-type-specific vulnerabilities. This provides a compelling mechanistic rationale for its multi-cancer therapeutic application and has the potential to advance the treatment paradigm from an organ-based classification toward one based on driver oncogenes. Despite its broad-spectrum anti-cancer efficacy, studies have identified challenges for the clinical translation of SS, including potential hepatotoxicity (40) and low bioavailability (41). Addressing these issues—specifically, reducing its liver toxicity and improving its bioavailability—is therefore a critical step toward its successful clinical application.

## Limitations and future perspectives

Despite these insights, our study has certain limitations. The network pharmacology predictions, while powerful, are computational in nature and require further extensive experimental validation. The current *in vitro* findings, particularly regarding the central role of SRC, need to be confirmed in *in vivo* models of BC, BLCA, and HCC. Moreover, the precise mechanism by which SS downregulates SRC expression—whether through direct transcriptional repression or post-translational regulation—remains to be elucidated. Future research should employ techniques such as kinase activity assays and Co-Immunoprecipitation (Co-IP) to dissect the exact molecular cascade. Additionally, investigating potential synergistic effects between SS and existing chemotherapeutic agents could pave the way for novel combination therapeutic strategies.

## Conclusion

In conclusion, our integrated strategy successfully bridged computational prediction with experimental validation, revealing that solasonine inhibits cancer progression in BC, BLCA, and HCC through a multi-target mechanism centered on the key protein SRC. This work not only elucidates the pharmacological basis of solasonine’s broad-spectrum anti-tumor activity but also identifies SRC and its associated network as a common therapeutic vulnerability across multiple cancer types. Our findings strongly support the continued development of solasonine as a promising multi-targeted agent or a strategic component in combination therapies for a range of malignancies.

## Data Availability

The datasets presented in this study can be found in online repositories. The names of the repository/repositories and accession number(s) can be found below: https://www.uniprot.org/, 1111.

## Supplementary material

Supplementary Figure 1 Network pharmacology of SS in CML. **(A)** Venn diagram of overlapping targets. **(B)** Key target proteins. **(C)** PPI network.

Supplementary Figure 2 Network pharmacology of SS in BCC. **(A)** Venn diagram of overlapping targets. **(B)** Key target proteins. **(C)** PPI network.

Supplementary Figure 3 Intersection of Top 10 hub genes across BC, BLCA, HCC, CML, and BCC.

Supplementary Figure 4 Prognostic impact of six core target genes on overall survival in CML.
