# Supplementary material for: Targeting SRC to mediate solasonine’s anti-cancer activity in hepatocellular carcinoma and its potential for multi-cancer therapy
Source: Front Oncol. 2026 Apr 27;16:1777213. doi: 10.3389/fonc.2026.1777213 (PMC13158681; doi:10.3389/fonc.2026.1777213)
Supplement: Supplementary Figure 1 — Network pharmacology of SS in CML. (A) Venn diagram of overlapping targets. (B) Key target proteins. (C) PPI network. [file SupplementaryFile1.pdf]

Supplementary Material

Fig. 1 Network pharmacology of SS in CML. (A) Venn diagram of overlapping targets. (B) Key target proteins. (C) PPI network.

Fig. 2 Network pharmacology of SS in BCC. (A) Venn diagram of overlapping targets. (B) Key target proteins. (C) PPI network.

Fig. 3 Intersection of Top 10 Hub Genes Across BC, BLCA, HCC, CML, and BCC

Fig. 4 Prognostic Impact of Six Core Target Genes on Overall Survival in CML

Supplementary Figure

Fig. 1

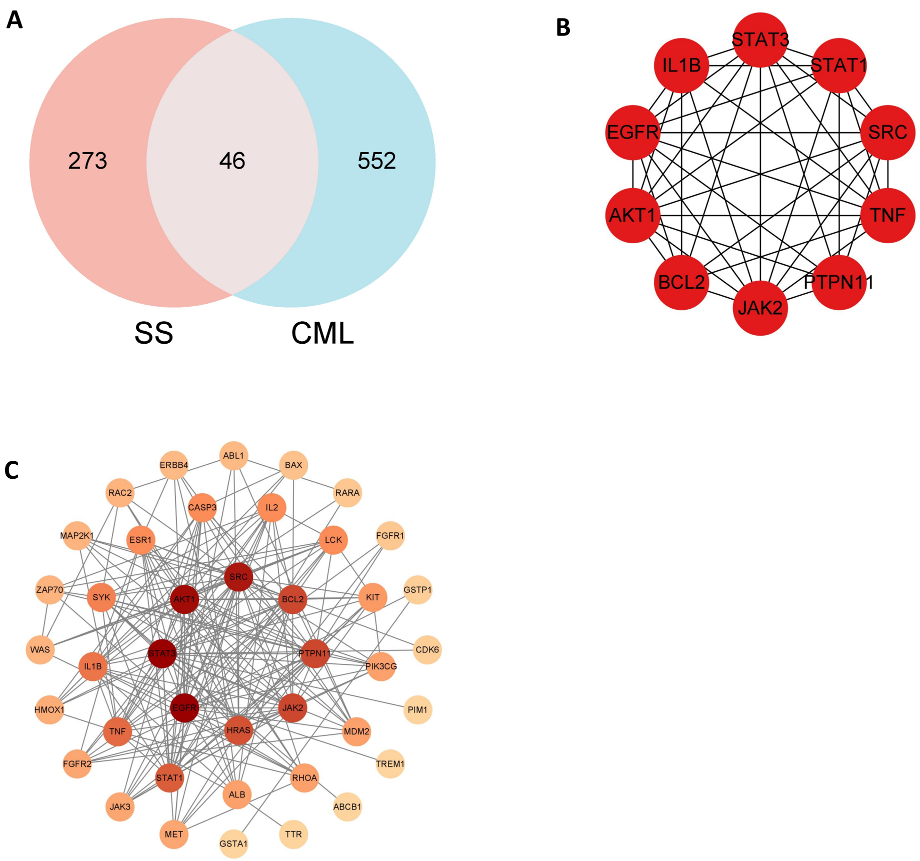

Fig. 2

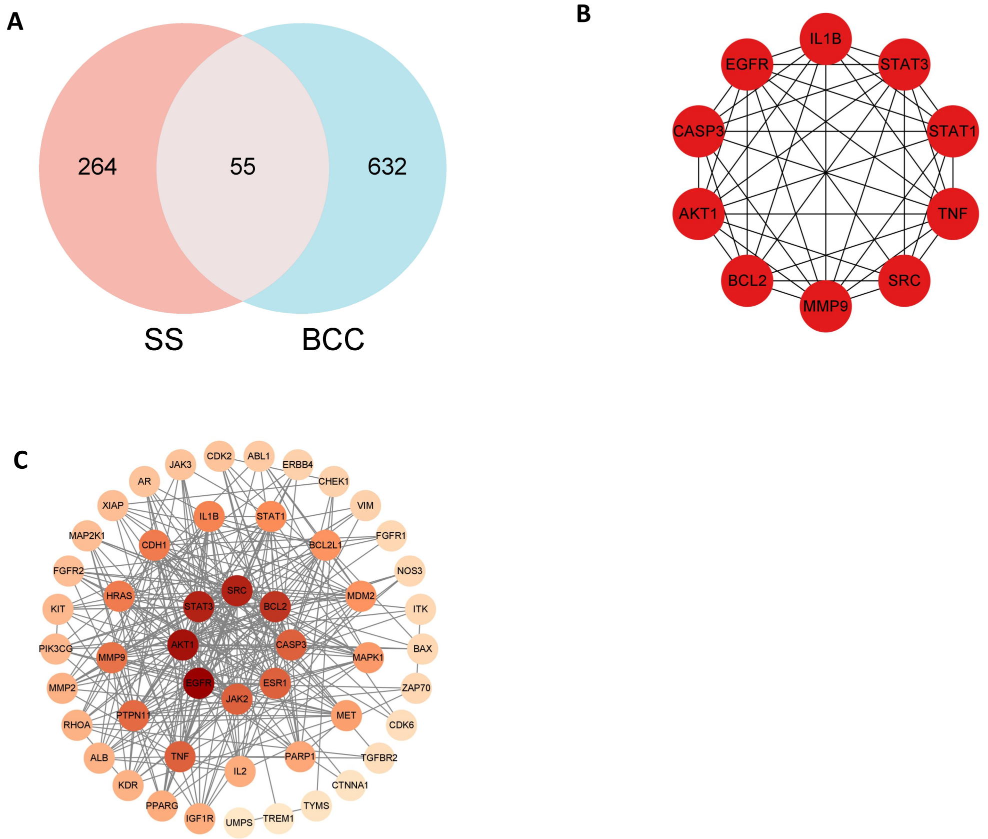
A
264
55
632
SS
BCC
B
IL1B
EGFR
STAT3
CASP3
STAT1
AKT1
TNF
BCL2
SRC
MMP9
C
CDK2
ABL1
JAK3
ERBB4
AR
CHEK1
XIAP
VIM
IL1B
STAT1
MAP2K1
CDH1
BCL2L1
FGFR1
FGFR2
NOS3
SRC
HRAS
MDM2
STAT3
BCL2
KIT
ITK
PIK3CG
AKT1
CASP3
BAX
MMP9
MAPK1
EGFR
ESR1
MMP2
ZAP70
JAK2
MET
PTPN11
RHOA
CDK6
TNF
PARP1
ALB
TGFBR2
IL2
KDR
CTNNA1
TYMS
PPARG
TREM1
IGF1R
UMPS

Fig. 3

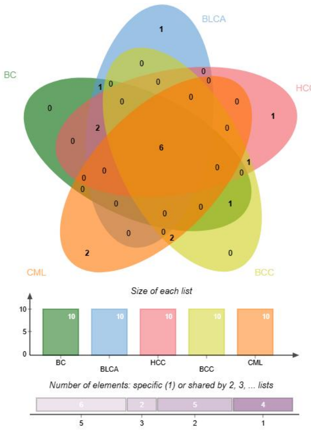
BLCA
BC
HCC
CML
BCC
Size of each list
Number of elements: specific (1) or shared by 2, 3, ... lists

Fig. 4

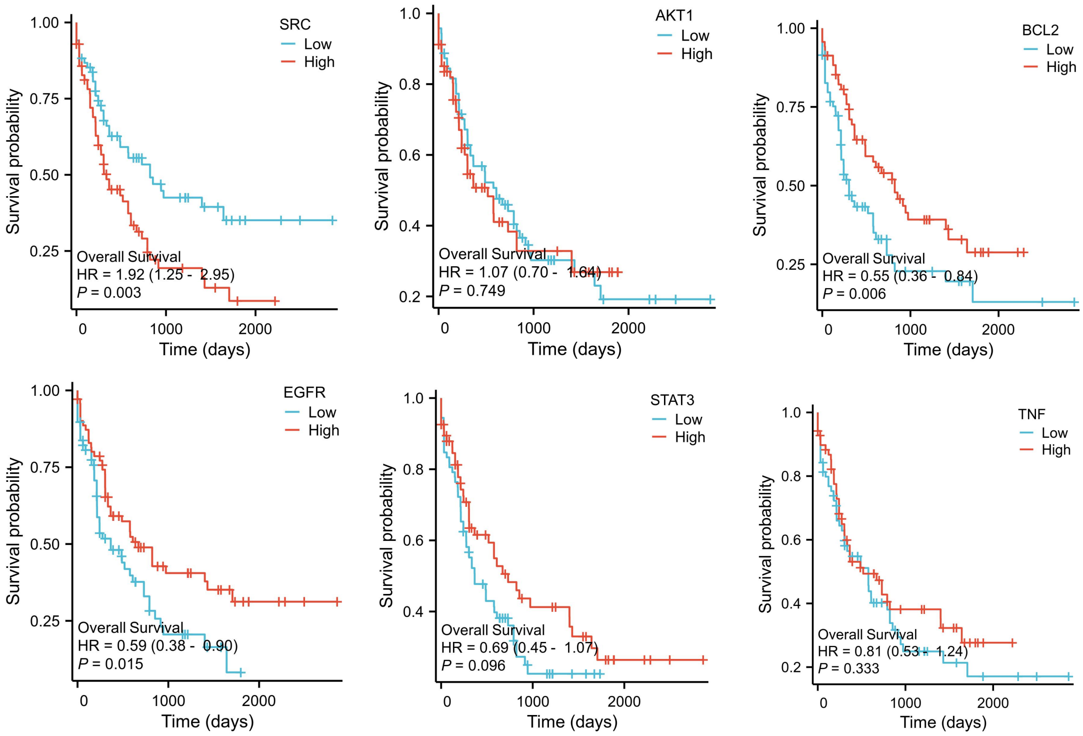

SRC
Low
High
Survival probability
Overall Survival
HR = 1.92 (1.25 - 2.95)
P = 0.003
Time (days)
AKT1
Low
High
Overall Survival
HR = 1.07 (0.70 - 1.64)
P = 0.749
BCL2
Low
High
Overall Survival
HR = 0.55 (0.36 - 0.84)
P = 0.006
EGFR
Low
High
Overall Survival
HR = 0.59 (0.38 - 0.90)
P = 0.015
STAT3
Low
High
Overall Survival
HR = 0.69 (0.45 - 1.07)
P = 0.096
TNF
Low
High
Overall Survival
HR = 0.81 (0.53 - 1.24)
P = 0.333
